# Amygdalar neurotransmission alterations in the BTBR mice model of idiopathic autism

**DOI:** 10.1038/s41398-024-02905-z

**Published:** 2024-04-17

**Authors:** Maria Bove, Maria Adelaide Palmieri, Martina Santoro, Lisa Pia Agosti, Silvana Gaetani, Adele Romano, Stefania Dimonte, Giuseppe Costantino, Vladyslav Sikora, Paolo Tucci, Stefania Schiavone, Maria Grazia Morgese, Luigia Trabace

**Affiliations:** 1 Department of Clinical and Experimental Medicine, University of Foggia, Via Napoli 20, 71122 Foggia, Italy; 2 Department of Physiology and Pharmacology “V. Erspamer”, Sapienza University of Rome, 00185 Rome, Italy; 3 Department of Pathology, Sumy State University, 40007 Sumy, Ukraine

**Keywords:** Molecular neuroscience, Neuroscience

## Abstract

Autism Spectrum Disorders (ASD) are principally diagnosed by three core behavioural symptoms, such as stereotyped repertoire, communication impairments and social dysfunctions. This complex pathology has been linked to abnormalities of corticostriatal and limbic circuits. Despite experimental efforts in elucidating the molecular mechanisms behind these abnormalities, a clear etiopathogenic hypothesis is still lacking. To this aim, preclinical studies can be really helpful to longitudinally study behavioural alterations resembling human symptoms and to investigate the underlying neurobiological correlates. In this regard, the BTBR T^+^ Itpr3^tf^/J (BTBR) mice are an inbred mouse strain that exhibits a pattern of behaviours well resembling human ASD-like behavioural features. In this study, the BTBR mice model was used to investigate neurochemical and biomolecular alterations, regarding Nerve Growth Factor (NGF) and Brain-Derived Neurotrophic Factor (BDNF), together with GABAergic, glutamatergic, cholinergic, dopaminergic and noradrenergic neurotransmissions and their metabolites in four different brain areas, i.e. prefrontal cortex, hippocampus, amygdala and hypothalamus. In our results, BTBR strain reported decreased noradrenaline, acetylcholine and GABA levels in prefrontal cortex, while hippocampal measurements showed reduced NGF and BDNF expression levels, together with GABA levels. Concerning hypothalamus, no differences were retrieved. As regarding amygdala, we found reduced dopamine levels, accompanied by increased dopamine metabolites in BTBR mice, together with decreased acetylcholine, NGF and GABA levels and enhanced glutamate content. Taken together, our data showed that the BTBR ASD model, beyond its face validity, is a useful tool to untangle neurotransmission alterations that could be underpinned to the heterogeneous ASD-like behaviours, highlighting the crucial role played by amygdala.

## Introduction

Autism Spectrum Disorders (ASD) are a group of neurodevelopmental disorders, generally diagnosed by three core behavioural symptoms, such as stereotyped repertoire, communication impairments and social withdrawal [1]. In addition, ASD are characterized by a wide range of additional symptoms, including cognitive dysfunctions, restricted interests, hyperactivity and impulsivity, thus showing comorbidities with different neuropsychiatric disorders, such as schizophrenia, attention-deficit/hyperactivity disorder, anxiety and obsessive-compulsive disorder [2, 3]. ASD prevalence has increased dramatically during the last decade, reaching the 1% estimation from the World Health Organization, that only accounts for approximately 16% of the global paediatric population; while European and US reports estimated around 1.4-2.5% in children of 8 years old on average based on population studies [4–7]. The complex etiopathogenesis underlying ASD onset and development is still unknown, since beyond the mostly hypothesized genetic and environmental involvement, multiple factors, regarding immune, dietary, metabolic and gastrointestinal systems, have recently been implicated [8, 9]. Considering that studies involving humans are often polluted by uncontrollable variables and biases and some contrasting results and inconsistencies in clinical evaluations have also been denounced, animal models can be really helpful to longitudinally study behavioural alterations resembling human symptoms in a translational way and to investigate the underlying neurobiological mechanisms [10, 11]. To this aim, the BTBR T^+^ Itpr3^tf^/J (BTBR) mice are an inbred mouse strain that well resembles the principal behavioural deficits of ASD [2, 12]. Among the different ASD mice models, the BTBR mouse strain shows robust and pronounced deficits in reciprocal social interactions, altered ultrasonic vocalization and repetitive stereotyped repertoire [13, 14]. In addition, BTBR mice display cognitive and emotional abnormalities to the psychiatric comorbidity of ASD [15, 16]. Considering their unique behavioural profile, BTBR mice might be a helpful tool to disentangle the neurobiological alterations that give rise to the heterogeneity of ASD symptoms, to identify putative biomarkers and, ultimately, to develop efficacious pharmacological treatments [17]. Regarding repetitive and restricted behaviours, BTBR mice were able to display both “lower-order” motor stereotypies and “higher-order” cognitive stereotypies [18], making this animal model suitable to study the neurobiological pathways related to these behavioural dysfunctions. Pharmacological treatments to ameliorate repetitive behaviours are mainly focused towards impulsivity and irritability, by using typical and atypical antipsychotics, thus they are employed in limited cases. Currently, there are no drugs approved to successfully treat ASD repetitive symptoms [19–22]. Hence, unravelling the burden of underlying mechanisms related to stereotypies would provide a valuable step forward to the development of novel pharmacological interventions to target ASD repetitive repertoire. Although the pathophysiology of ASD is still cryptic, several imaging and *post-mortem* studies reported the crucial involvement of corticostriatal and limbic circuits, by showing cortical, hippocampal and amygdalar anatomical abnormalities and functional alterations [23–27].

Indeed, a number of genes related to ASD are known to alter the neural structure, function and connectivity, as well as neurotransmission and neurotropism [28, 29]. In this regard, the neurotrophin family, notably Brain-Derived Neurotrophic Factor (BDNF) and Nerve Growth Factor (NGF), plays a pivotal role in neurodevelopmental processes which are atypical in ASD pathology [30]. In particular, BDNF, being an essential actor for the proper cerebral development and importantly associated to synaptic plasticity, has been recently proposed as a possible diagnostic marker in children suffering from ASD and different line of evidence have reported its involvement in ASD onset and development [31–33]. As regarding Nerve Growth Factor (NGF), such neurotrophin plays a key role in the regulation of nerve-cell growth, survival and differentiation, being markedly expressed in the central nervous system, particularly in the cerebral cortex, hippocampus (HIPP) and amygdala (AMY) [34]. Research linking NGF and ASD is limited. Few studies report an involvement of the NGF signalling pathway in the pathogenesis of ASD [30, 35], also considering the critical role of this neurotrophin in the immune modulation and in the induction of the release of different neuropeptides and neurotransmitters [36], such as glutamate and dopamine (DA) [37]. On the other hand, cathecolamines are known to increase NGF content [38], thus resulting in a continuous crosstalk between these two pathways. In this regard, cathecolamines, such as DA and noradrenaline (NA) are known to play a pivotal role in the modulation of executive functions, attention, impulsivity and emotional state, which are all processes disrupted in ASD [39, 40]. Indeed, recent studies report a possible implication of DA neurotransmission and metabolism in ASD development [41–43] while a number of evidence demonstrated that neuropathological changes related to the noradrenergic system often occur in ASD [44–46]. In addition, the imbalance of the excitatory-inhibitory synaptic transmission has also been linked to ASD pathology [47]. In this regard, alterations of glutamate and gamma-aminobutyric acid (GABA) receptors expression have been reported in *post-mortem* brains of ASD patients [48] and a decrease in GABA levels have been observed in different brain regions of ASD children [49]. Furthermore, brain regions with dense cholinergic innervation, such as prefrontal cortex (PFC), hippocampus (HIPP) and amygdala (AMY), are linked to social cognition, thus resulting crucially involved in ASD pathophysiology [50]. In this regard, it has been reported that low acetylcoline (ACh) levels in mice displayed reduced social interactions and that, by administrating acetylcholinesterase inhibitors, such impairment might be relieved [51]. Converging evidence have revealed that ASD patients show an atypical social brain circuitry and that, in this important network, AMY is one of the regions with major interest [52]. In this regard, it has been reported that ASD patients showed reduced connectivity between AMY and PFC that was correlated with the severity of social dysfunctions [53]. In addition, a weaker functional connectivity was also found between AMY and occipital cortex [54], suggesting that such region, being the core of social brain network, deserves deeper investigations.

To summarize, a number of molecular alterations, in terms of neurotrophin, cathecolamines and aminoacids have been reported in both animal and human ASD studies, and, from an anatomical point of view, tendency towards overgrowth of PFC and AMY have been detected in individuals with ASD compared to neurotypical controls [55–57]. Moreover, data from structural magnetic resonance imaging highlighted widespread cerebral abnormalities in ASD patients, that involve total brain volume, fronto-parieto-temporal and cerebellar regions [58]. Nonetheless the above mentioned alterations, ASD continue to be purely defined by behavioural dysfunctions, thus to unravel the underpinning mechanisms beyond behavioural traits still represents a demanding task.

To this aim, we employed the BTBR mice model to investigate neurochemical and biomolecular alterations underpinning ASD behavioural dysfunctions. In particular, we quantified NGF and BDNF expression levels, together with GABAergic, glutamatergic, dopaminergic, noradrenergic and cholinergic neurotransmissions in PFC, HIPP, AMY and hypothalamus (HYP).

## Materials and Methods

### Animals

In this study, we focused on male sex since ASD shows a male prevalence, being males three times more diagnosed than females [59]. A total of 20 (2–3 per litter, randomly distributed) 10-weeks-old male mice, C57/BL6J (BL6) (Envigo, San Pietro al Natisone, Italy) and BTBR (Charles-River Italia, Milan, Italy), were used in this experimental study. They were raised at constant room temperature (22 ± 1 °C) with relative humidity (55 ± 5%), under a 12 h light/dark cycle (lights on from 7:00 AM to 7:00 PM) and free access to water and food. All experiments on animals and their care were carried out in accordance with the institutional guidelines of the Italian Ministry of Health (D.Lgs. n. 26/2014), the Guide for the Care and Use of Laboratory Animals: Eight Edition, the Guide for the Care and Use of Mammals in Neuroscience and Behavioral Research (National Research Council, 2004), the Directive 2010/63/EU of the European Parliament and of the Council of 22 September 2010 on the protection of animals used for scientific intents, as defined by Animal Research: Reporting of In Vivo Experiments (ARRIVE) guidelines 2.0. The experimental protocol was approved by the Italian Ministry of Health (protocol nr. B2EF8.24). Animals’ health state was checked daily during experimental period. Moreover, to accomplish the 3 R’s principles, we performed the behavioural test battery on the same group of animals; in order to reduce as much as possible the number of animals used and every procedure was performed with the aim to minimize their suffering.

### Battery of behavioural tests

#### Hole board test

During day 1, BTBR and BL6 animals performed the Hole Board task. This test was carried out in a wooden box (40 × 40 x 35 cm) having 16 holes with a diameter of 3 cm on the ground and placed 5 cm from the floor, as previously described [60]. The test lasted 10 min and an automatic counter registered the number of times the animals poking the hole for a duration of at least 1 sec, reported as number of poking holes.

#### Open Field test

During day 2, the Open Field test was carried out. The test was performed according [61]. Briefly, the animals were left to explore an open field arena (40 × 40 x 35 cm) for 5 min. ANY-maze tracking software version 7 (Ugo Basile-Varese, Gemonio, Italy) recorded and analyzed the locomotory activity of each mouse by measuring the distance travelled, the duration and the frequency of freezing behaviour and the time spent in the center and in the wall of the arena. Between one test and another, a solution at 70% of ethanol was used to clean arena floor and avoid inter-assay bias.

#### Social Interaction test

During day 3, the Social Interaction test was performed, as previously described [62]. The animals, after 2 days of individual housing, were left in the same large box used for the Open Field arena and let free to explore one plastic object, one paper cylinder, one ball and an unfamiliar stimulus mouse (same strain, sex and age of subject mouse) for 5 min. A camera recorded the test and a blind observer scored the frequency and duration of social behaviours from an investigative and affiliative point of view and also frequency and duration of non-social behaviour, considering the attitude towards objects, like sniffing, exploring and playing with the objects. [63, 64].

#### Elevated Zero Maze test

During day 4, the Elevated Zero Maze test was carried out, as previously described [65]. Precisely, a maze built in black acrylic in a circular track 10 cm wide, 105 cm in diameter and 72 cm high was used. Two opposed closed quadrants and two opposed open quadrants with black acrylic walls 28 cm high composed the maze. On the test day the animal was placed at a casually chosen boundary between an open and a closed zone, facing the closed area. After each trial, the maze was cleaned with a 70% ethanol solution. The test lasted 5 min and a blind observer evaluated the time spent in the open and in the closed corridors, expressed in seconds.

### *Post-mortem* tissues analyses

After behavioural tests, the mice were sacrificed by cervical dislocation and brains were randomly divided for ex vivo analysis. PFC, HIPP, HYP and AMY were removed from brains, in accordance with the mouse brain atlas of Paxinos and Franklin, and frozen, stored at −80 °C for subsequent analyses. To perform biomolecular studies, the tissues were homogenated and diluted 1:10 w/v in PBS buffer with 1:100 protease and phosphatase inhibitor (HALT inhibitors, Thermo Fisher Scientific, Cleveland, OH, USA) at 4 °C; while for neurochemical analyses, the samples were homogenated and diluted 1:10 w/v in perchloric acid 0.1 M at 4 °C. In both cases, after dilution, a centrifuge at 10.000 x *g* at 4 °C for 10 min was carried out and the supernatants were analysed.

### Western blotting quantification

In this procedure, protein extracts were generated from fresh frozen PFC, HIPP and AMY tissues following samples homogenization with a Halt™ Protease and Phosphatase Inhibitor Single-Use Cocktail, EDTA-Free (Thermo Fisher Scientific, Cleveland, OH, USA). Subsequently, lysates were measured for total protein concentration using a Pierce™ BCA protein assay kit (Thermo Fisher Scientific, Cleveland, OH, USA) and the Multiskan™ FC Microplate spectrophotometer (Thermo Fisher Scientific, USA) at 570 nm. For SDS-PAGE the total amount of samples protein (forty µg) loaded in to the 4–15% Mini-PROTEAN™ TGX Stain-Free™ Protein Gels (Bio-Rad Laboratories Inc, Segrate (MI), Italy) for electrophoresis and then transferred on the Nitrocellulose membrane (Bio-Rad Laboratories Inc, Segrate (MI), Italy) by Pierce™ Power Blotter (Thermo Fisher Scientific, Cleveland, OH, USA). Then, the membranes were blocked in the 5% skimmed milk for 1 h at room temperature followed the incubation with rabbit monoclonal antibodies against NGF (ab52918; 1:1000, Abcam, Cambridge, UK), BDNF (ab226843; 1:1000, Abcam, Cambridge, UK) and mouse monoclonal antibodies against β-actin (ab8226; 1:1000, Abcam, Cambridge, UK) overnight at 4 °C. In add to this, the incubation (1 h at room temperature) with horseradish peroxidase-conjugated specific (Goat anti-rabbit (ab6721; 1:5000, Abcam, UK) and goat anti-mouse (ab205719; 1:5000, Abcam, UK)) secondary antibodies were used. Clarity™ Western ECL Substrate (Bio-Rad Laboratories Inc, Segrate (MI), Italy) was used for protein bands visualization. ChemiDoc™ XRS+ system (Bio-Rad Laboratories Inc, Segrate (MI), Italy) was used to detect chemiluminescence and ImageJ software (version 1.52a; National Institutes of Health, USA) was utilized to quantify the optical densities of the bands that were then normalized versus bands of β-actin.

### Neurochemical quantifications

The neurochemical quantifications were carried out in PFC, HIPP and AMY of BTBR and BL6 animals. In particular DA, NA, 3,4-dihydroxyphenylacetic acid (DOPAC), homovanillic acid (HVA) were measured by using high-performance liquid chromatography coupled with an electrochemical detector (Ultimate ECD, Dionex Scientific, Milan, Italy). LC18 reverse phase column (Kinetex, 150 mm × 3.0 mm, ODS 5 µm; Phenomenex, Castel Maggiore-Bologna, Italy) was utilized to separate the catecholamines that were detected by a thin-layer amperometric cell (Dionex, ThermoScientific, Milan, Italy) with a 5-mm diameter glassy carbon electrode by using 400 mV as working potential vs. Pd. An aqueous buffer (pH 3.0) composed of 75 mM NaH2PO4, 1.7 mM octane sulfonic acid, 0.3 mM EDTA, acetonitrile 10%, was used as mobile phase and an isocratic pump (Shimadzu LC-10 AD, Kyoto, Japan) worked at 0.7 ml·min-1 as flow rate. To perform the data acquisition and integration, Chromeleon software (version 6.80, Dionex, Thermo Scientific, San Donato Milanese, Italy) was utilized. Moreover, GABA and glutamate amounts were quantified by high-performance liquid chromatography with fluorescence detection, after derivatization with ophthalaldehyde/mercaptopropionic acid (emission length, 460 nm; excitation length, 340 nm) by using an OD column (Kinetex, 150 mm×3.0 mm, ODS 5 µm; Phenomenex, Castel Maggiore-Bologna, Italy). The mobile phase used was a gradient phase of 50 mM sodium acetate buffer, pH 6.95, with methanol increasing linearly from 2 to 30% (v/v) over 40 min. A pump (JASCO, Tokyo, Japan) maintained the flow rate at 0.5 ml/min and the Borwin software (version 1.50; Jasco) was used to analyze the results. Results, after being normalized for total area weight, were expressed as concentration/mg of tissue.

### Acetylcholine (ACh) assay

The levels of ACh were quantified in tissue homozenaized by using the commercially available kit (Catalog Number MAK435, Sigma-Aldrich, Milano, Italy), following the manufacturer’s instructions. Tissue has been prepared by homogenization in cold 1× PBS and centrifugation (5 min at 14,000 × *g*). In the assay utilized, acetylcholine is hydrolysed by acetylcholinesterase to choline which is oxidized by choline oxidase to betaine and H2O2. H2O2 reacts with a specific dye to form a colored product. The color intensity was analysed by the Multiskan™ FC Microplate spectrophotometer (Thermo Fisher Scientific, USA) at 570 nm and it is directly proportional to the acetylcholine concentration in the sample. Results were expressed in accordance to the linear detection range for the acetylcholine assay method (10-200 μM). Each sample analysis was performed in duplicate to avoid intra-assay variations.

### Blindness of the study

For each test, scoring process and analysis, the experimenters were blind with respect to the experimental groups.

### Statistical analyses

Sample size calculation has been performed a priori by using G power software. Statistical analyses were carried out by GraphPad Prism software (version 9.5.0; San Diego, CA, USA). In particular, Shapiro-Wilk test for normality and ROUT method to identify statistical outliers were performed for each group. Subsequently, Unpaired Student’s *t-*test two-sided, with Welch’s correction when needed, was used to analyze data that were expressed as mean ± standard error of the mean (SEM). Correlation between behavioural outcomes and ex vivo results were analyzed by using Pearson correlation. Differences between groups were considered significant with a *P* value less than 0.05.

## Results

### BTBR mice showed increased repetitive behaviour and novelty-induced hyperlocomotion

In order to evaluate repetitive behaviours, we performed the Hole Board task. Our results showed that BTBR mice reported a significant increase in the number of poking holes compared to control mice (Supplementary Fig. 1A, Unpaired Student’s *t*-test, *P* < 0.001 BTBR vs. BL6). Furthermore, the locomotor activity in a novel environment was investigated in the Open Field paradigm. We observed an increase of the distance travelled in meters in the arena, index of novelty-induced hyperlocomotion, in BTBR mice compared with control animals (Supplementary Fig. 1B, Unpaired Student’s *t*-test, *P* < 0.0001 BTBR vs. BL6). Furthermore, BTBR also showed a decrease in freezing duration (Supplementary Fig. 1C, Unpaired Student’s *t*-test, *P* < 0.05 BL6 vs. BTBR) and freezing frequency (Supplementary Fig. 1D, Unpaired Student’s *t*-test, *P* < 0.05 BL6 vs. BTBR) compared with BL6 animals.

### BTBR mice showed reduced social behaviours and increased non-social behaviours

To investigate social dysfunctions, the Social Interaction test was performed. During Social Interaction test, BTBR mice performed significantly less social duration time (sec) compared to BL6 mice (Supplementary Fig. 2A, Unpaired Student’s *t*-test, *P* < 0.05 BTBR vs. BL6). Moreover, the time (sec) spent performing non-social interactions in BTBR animals was significantly increased compared to controls (Supplementary Fig. 2B, Unpaired Student’s *t*-test, *P* < 0.0001 BTBR vs. BL6). As regarding the frequency of such behaviours, BTBR social frequency measurements were significantly reduced (Supplementary Fig. 2C, Unpaired Student’s *t*-test, *P* < 0.05 BL6 vs. BTBR), while there was a significant enhancement in non-social frequency compared to BL6 animals (Supplementary Fig. 2D, Unpaired Student’s *t*-test, *P* < 0.0001 BTBR vs. BL6).

### BTBR mice showed reduced anxiety-like behaviours

To evaluate the anxiety-like behaviours, we used the Elevated Zero Maze and the Open Field tests. We found that BTBR animals showed an increase in the time spent exploring the open corridors compared to controls (Supplementary Fig. 3A, Unpaired Student’s *t*-test, *P* < 0.001 BTBR vs. BL6). Accordingly, the time spent in closed quadrants of BTBR mice was significantly decreased compared to BL6 (Supplementary Fig. 3B, Unpaired Student’s *t*-test, *P* < 0.001 BTBR vs. BL6). Moreover, results from Open field test showed that the time spent in the center in BTBR animals was significantly increased compared to control animals (Supplementary Fig. 3C, Unpaired Student’s *t*-test, *P* < 0.0001 BTBR vs. BL6), while the time spent in the wall was a significantly reduced, respectively (Supplementary Fig. 3D, Unpaired Student’s *t*-test, *P* < 0.0001 BTBR vs. BL6).

Subsequently, we performed ex vivo analyses in PFC, HIPP, HYP and AMY tissues to quantify NA, DA and DA metabolites (DOPAC and HVA) levels, together with Acetylcholine (ACh), Glutamate and GABA, and NGF and BDNF expression levels in both experimental groups.

### BTBR mice showed reduced NA, ACh and GABA levels in PFC

**Fig. 1:**
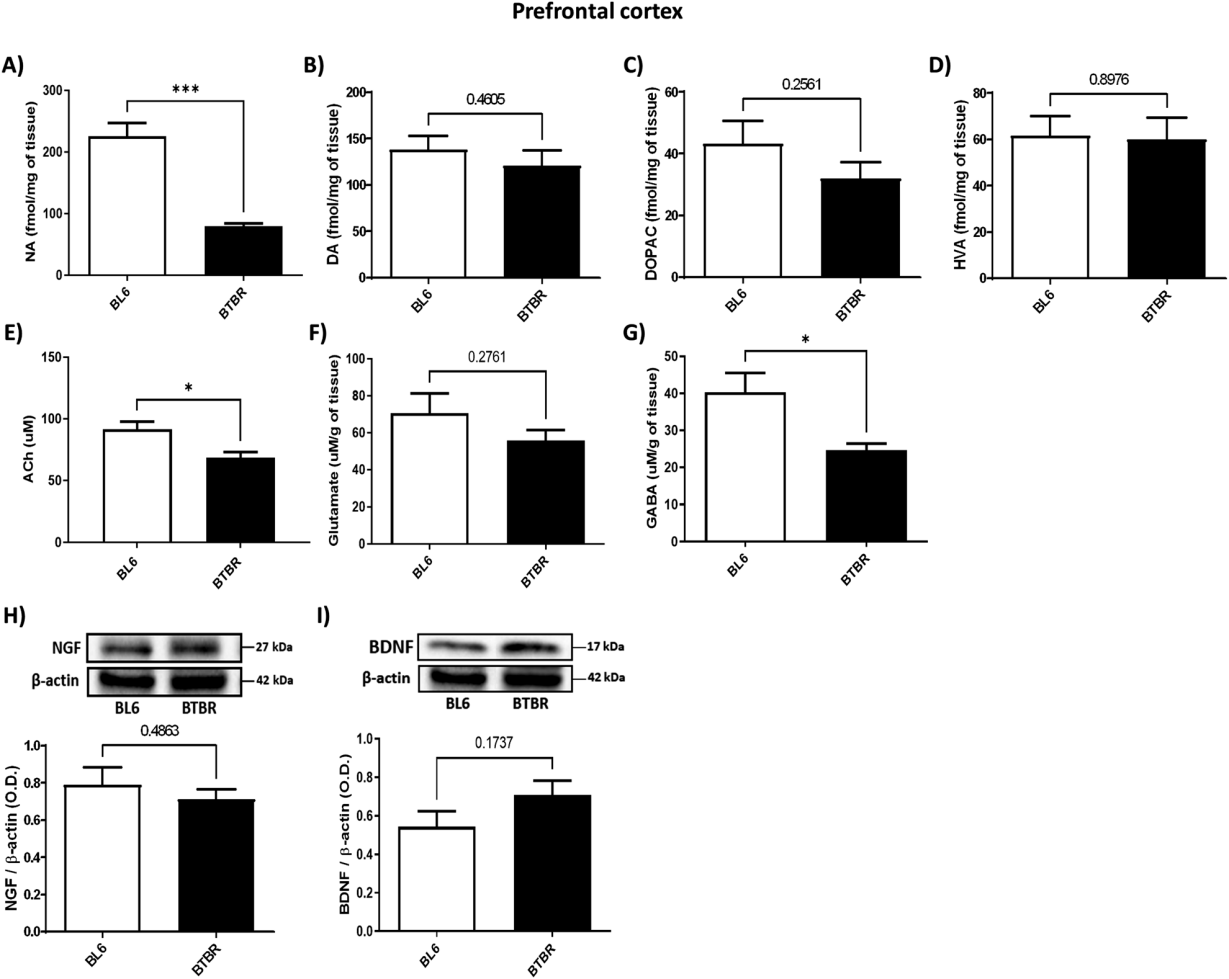
NA, DA, DOPAC, HV, Ach, Glutamate, GABA, NGF and BDNF levels in PFC of BTBR (black bar) and BL6 (white bar) mice. **A** NA levels (fmol/mg) of BL6 (*n* = 5) and BTBR (*n* = 5) mice. Unpaired Student’s *t*-test, ***P = 0.0008 BTBR vs. BL6; **B** DA amount (fmol/mg) in the PFC of BL6 (*n* = 5) and BTBR (*n* = 5) mice. Unpaired Student’s t-test, n.s.; **C** DOPAC levels (fmol/mg) in the PFC of BL6 (*n* = 5) and BTBR (*n* = 5) mice. Unpaired Student’s *t*-test, n.s.; **D** HVA levels (fmol/mg) in the PFC of BL6 (*n* = 5) and BTBR (*n* = 5) mice. Unpaired Student’s *t*-test, n.s.; **E** Ach levels (uM) in the PFC of BL6 (*n* = 5) and BTBR (*n* = 4) mice. Unpaired Student’s *t*-test, *P = 0.0176 BTBR vs. BL6; **F** Glutamate levels (uM/mg) of BL6 (*n* = 5) and BTBR (*n* = 5) mice. Unpaired Student’s t-test, n.s.; **G** GABA levels (uM/mg) of BL6 (*n* = 5) and BTBR (*n* = 5) mice. Unpaired Student’s *t*-test, *P = 0.0290 BTBR vs. BL6; **H** NGF expression levels of BTBR (*n* = 5) and BL6 (*n* = 4). Unpaired Student’s *t*-test, n.s. Quantification of the optical band density of NGF normalized for optical band density of β-actin housekeeping gene; **I** BDNF expression levels of BTBR (*n* = 5) and BL6 (*n* = 4). Unpaired Student’s *t*-test, n.s. Quantification of the optical band density of BDNF normalized for optical band density of β-actin housekeeping gene.

Our results reported a significantly decrease of NA levels in the PFC of BTBR animals compared to BL6 (Fig. 1A, Unpaired Student’s *t*-test, *P* < 0.0001 BTBR vs. BL6), together with ACh and GABA levels, respectively (Figs. 1E, G, Unpaired Student’s *t*-test, *P* < 0.05 BTBR vs. BL6). As regarding DA, DOPAC, HVA, NGF, BDNF and glutamate content, our results did not show any difference between the two groups (Fig. 1, Unpaired Student’s *t*-test, n.s.).

### BTBR mice showed enhanced DOPAC and decreased NGF, BDNF and GABA levels in HIPP

**Fig. 2:**
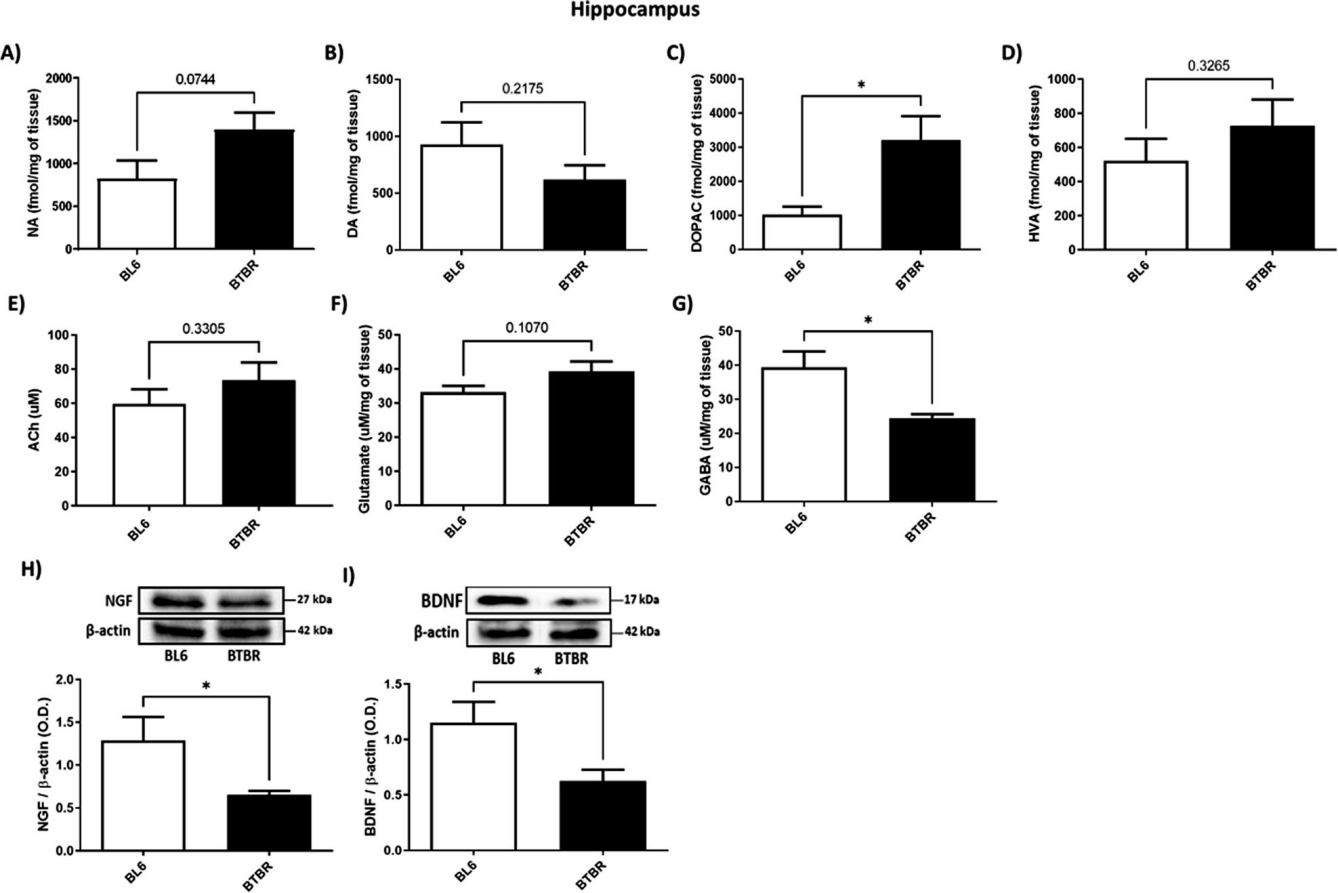
NA, DA, DOPAC, HV, Ach, Glutamate, GABA, NGF and BDNF levels in HIPP of BTBR (black bar) and BL6 (white bar) mice. **A** NA amount (fmol/mg) of BL6 (*n* = 5) and BTBR (*n* = 5) mice. Unpaired Student’s t-test, n.s.; **B** DA levels (fmol/mg) of BL6 (*n* = 5) and BTBR (*n* = 5) mice. Unpaired Student’s t-test, n.s.; **C** DOPAC amount (fmol/mg) of BL6 (*n* = 5) and BTBR (*n* = 5) mice. Unpaired Student’s *t*-test, *P = 0.0323 BTBR vs. BL6; **D** HVA amount (fmol/mg) of BL6 (*n* = 5) and BTBR (*n* = 5) mice. Unpaired Student’s *t*-test, n.s.; **E** Ach levels (uM) of BL6 (*n* = 5) and BTBR (*n* = 4) mice. Unpaired Student’s *t*-test, n.s.; **F** Glutamate levels (uM/mg) of BL6 (*n* = 5) and BTBR (*n* = 5) mice. Unpaired Student’s *t*-test, n.s.; **G** GABA levels (uM/mg) of BL6 (*n* = 5) and BTBR (*n* = 5) mice. Unpaired Student’s t-test, *P = 0.0221 BTBR vs. BL6; **H** NGF expression levels of BTBR (*n* = 5) and BL6 (*n* = 4). Unpaired Student’s *t*-test, *P = 0.0361 BTBR vs. BL6. Quantification of the optical band density of NGF normalized for optical band density of β-actin housekeeping gene; **I** BDNF expression levels of BTBR (*n* = 5) and BL6 (*n* = 4). Unpaired Student’s t-test, *P = 0.0352 BTBR vs. BL6. Quantification of the optical band density of BDNF normalized for optical band density of β-actin housekeeping gene.

As regards HIPP, we did not find any difference in NA, DA, HVA, ACh and glutamate levels between BTBR and BL6 mice (Fig. 2, Unpaired Student’s *t*-test, n.s.). Moreover, analysis of DOPAC content reported a significant increase in BTBR animals compared to controls (Fig. 2C, Unpaired Student’s *t*-test, *P* < 0.05 BTBR vs. BL6), while NGF and BDNF expression levels, together with GABA content were decreased (Figs. 2G–I, Unpaired Student’s *t*-test, *P* < 0.05 BTBR vs. BL6).

### BTBR mice did not show any difference in HYP

**Fig. 3:**
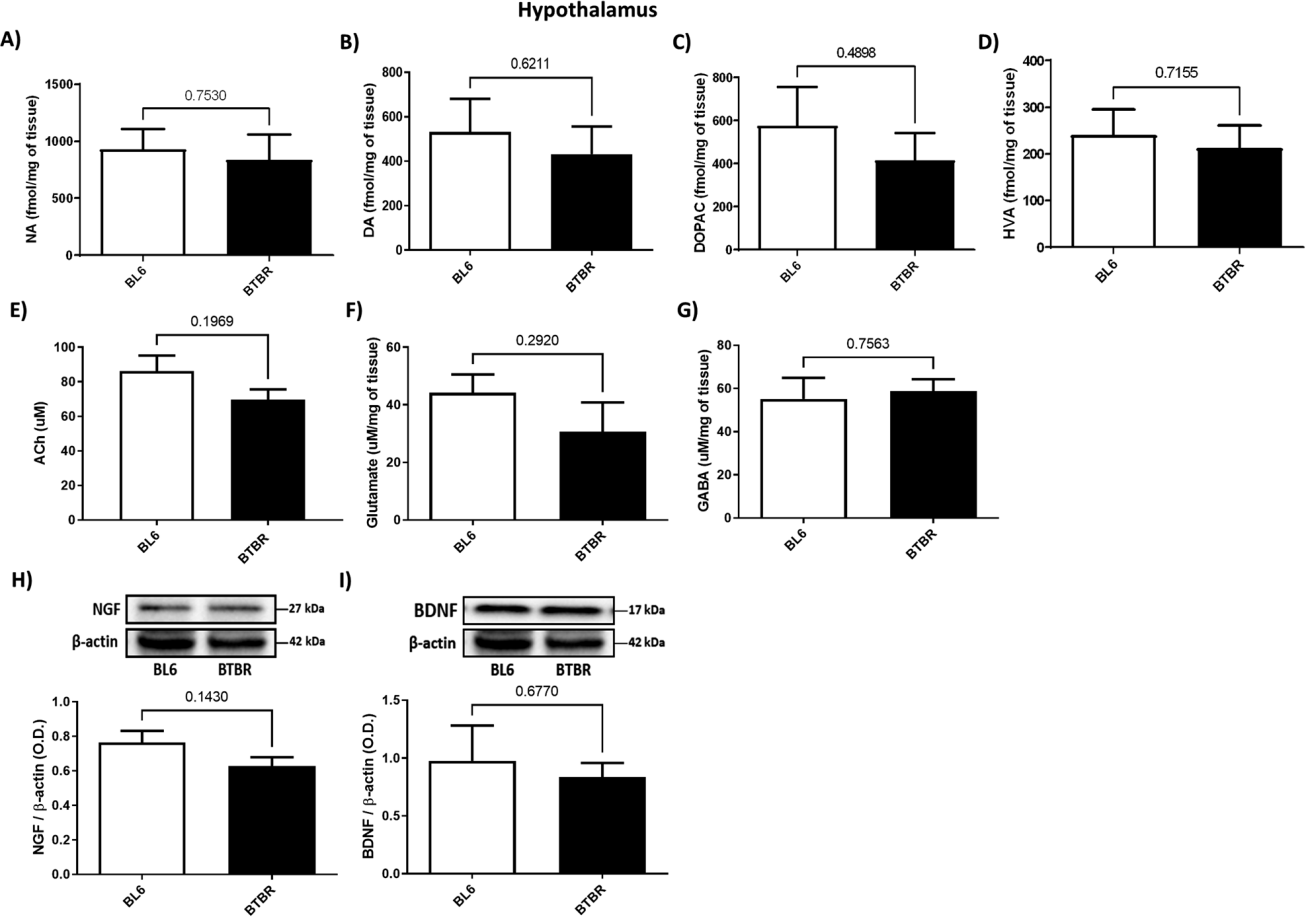
NA, DA, DOPAC, HV, Ach, Glutamate, GABA, NGF and BDNF levels in HYP of BTBR (black bar) and BL6 (white bar) mice. **A** NA amount (fmol/mg) of BL6 (*n* = 5) and BTBR (*n* = 5) mice. Unpaired Student’s t-test, n.s.; **B** DA levels (fmol/mg) of BL6 (*n* = 5) and BTBR (*n* = 5) mice. Unpaired Student’s t-test, n.s.; **C** DOPAC amount (fmol/mg) of BL6 (*n* = 5) and BTBR (*n* = 5) mice. Unpaired Student’s t-test, n.s.; **D** HVA amount (fmol/mg) of BL6 (*n* = 5) and BTBR (*n* = 5) mice. Unpaired Student’s *t*-test, n.s.; **E** Ach levels (uM) of BL6 (*n* = 5) and BTBR (*n* = 4) mice. Unpaired Student’s *t*-test, n.s.; **F** Glutamate levels (uM/mg) of BL6 (*n* = 5) and BTBR (*n* = 5) mice. Unpaired Student’s *t*-test, n.s.; **G** GABA levels (uM/mg) of BL6 (*n* = 5) and BTBR (*n* = 5) mice. Unpaired Student’s t-test, n.s.; **H** NGF expression levels of BTBR (*n* = 5) and BL6 (*n* = 4). Unpaired Student’s *t*-test, n.s. Quantification of the optical band density of NGF normalized for optical band density of β-actin housekeeping gene; **I** BDNF expression levels of BTBR (*n* = 5) and BL6 (*n* = 4). Unpaired Student’s *t*-test, n.s. Quantification of the optical band density of BDNF normalized for optical band density of β-actin housekeeping gene.

Concerning HYP analysis, no differences in NA, DA, DOPAC, HVA, ACh, NGF, BDNF, glutamate and GABA levels were found between groups (Fig. 3, Unpaired Student’s *t*-test, n.s.).

### BTBR mice showed reduced DA, NA, ACh, NGF and GABA and increased DA metabolism and glutamate content in AMY

**Fig. 4:**
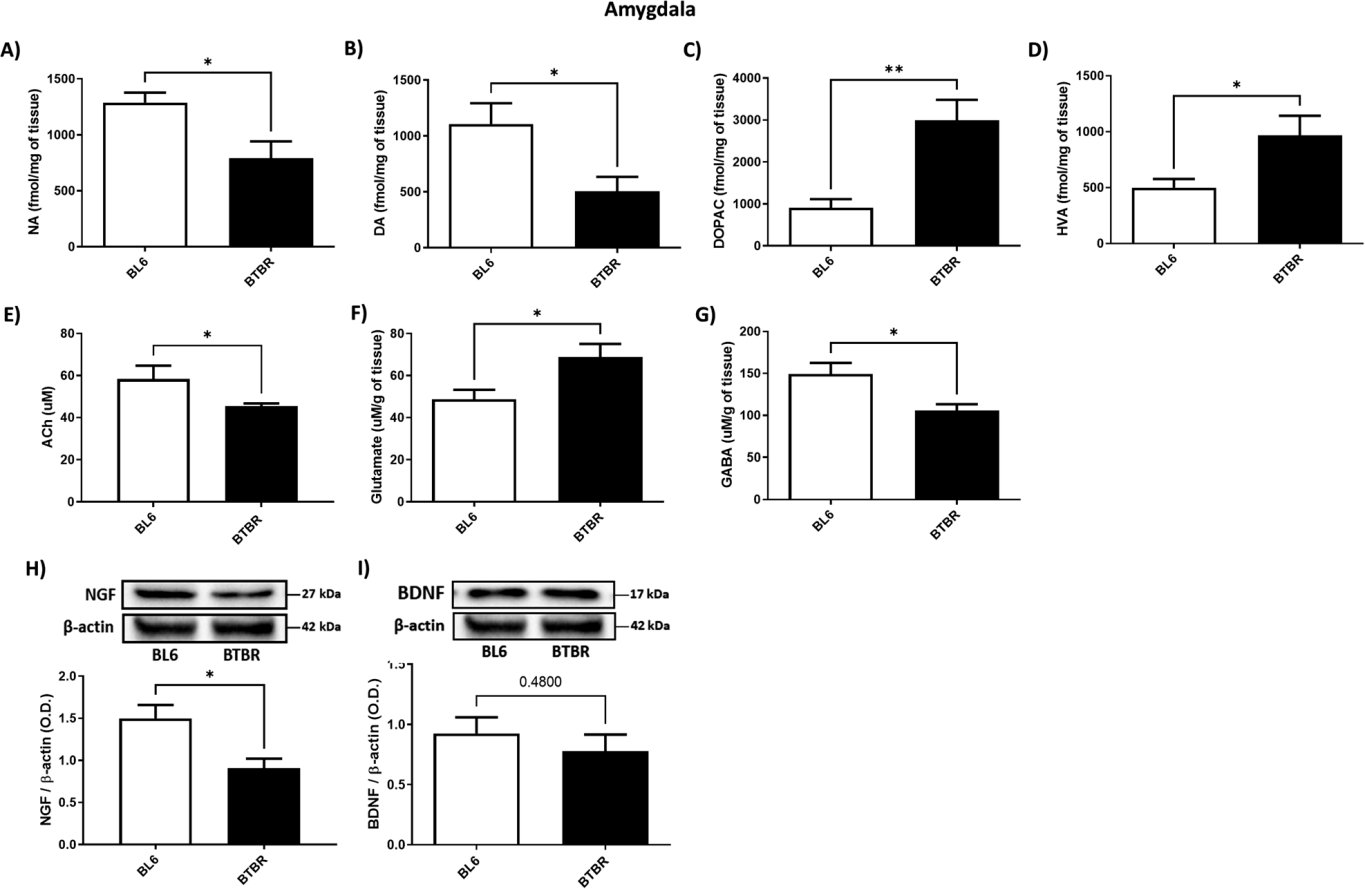
NA, DA, DOPAC, HV, Ach, Glutamate, GABA, NGF and BDNF levels in AMY of BTBR (black bar) and BL6 (white bar) mice. **A** NA levels (fmol/mg) of BL6 (*n* = 5) and BTBR (*n* = 5) mice. Unpaired Student’s *t*-test, *P = 0.0221 BTBR vs. BL6; **B** DA levels (fmol/mg) of BL6 (*n* = 5) and BTBR (*n* = 5) mice. Unpaired Student’s *t*-test, *P = 0.0266 BTBR vs. BL6; **C** DOPAC levels (fmol/mg) of BL6 (*n* = 5) and BTBR (*n* = 5) mice. Unpaired Student’s *t*-test, **P = 0.0100 BTBR vs. BL6; **D** HVA levels (fmol/mg) of BL6 (*n* = 5) and BTBR (*n* = 5) mice. Unpaired Student’s *t*-test, *P = 0.0469 BTBR vs. BL6; **E** Ach levels (uM) of BL6 (*n* = 5) and BTBR (*n* = 4) mice. Unpaired Student’s *t*-test, *P = 0.0286 BTBR vs. BL6; **F** Glutamate levels (uM/mg) of BL6 (*n* = 5) and BTBR (*n* = 5) mice. Unpaired Student’s *t*-test, *P = 0.0366 BTBR vs. BL6; **G** GABA levels (uM/mg) of BL6 (*n* = 5) and BTBR (*n* = 5) mice. Unpaired Student’s *t*-test, *P = 0.0244 BTBR vs. BL6; **H** NGF expression levels of BTBR (*n* = 5) and BL6 (*n* = 4). Unpaired Student’s *t*-test, *P = 0.0167 BTBR vs. BL6. Quantification of the optical band density of NGF normalized for optical band density of β-actin housekeeping gene; **I**) BDNF expression levels of BTBR (*n* = 5) and BL6 (*n* = 4). Unpaired Student’s *t*-test, n.s. Quantification of the optical band density of BDNF normalized for optical band density of β-actin housekeeping gene.

Our results showed that NA, DA, ACh, NGF and GABA levels in AMY were substantially reduced in BTBR compared to BL6 animals (Fig. 4, Unpaired Student’s *t*-test, *P* < 0.05 BTBR vs. BL6). In addition, DOPAC, HVA and glutamate content in AMY of BTBR animals showed a significant enhancement compared to controls (Figs. 4C, D and F, Unpaired Student’s *t*-test, *P* < 0.001, *P* < 0.05 BTBR vs. BL6), while BDNF did not exhibit any differences (Fig. 4I, Unpaired Student’s *t*-test, n.s.).

### BTBR mice showed negative correlation between repetitive behaviours and hyperlocomotion with amygdalar NA levels and between non social behaviours and amygdalar GABA amount

**Fig. 5:**
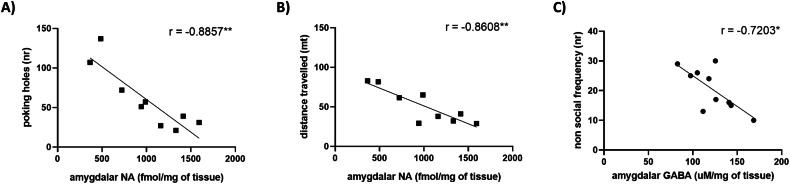
Correlation between repetitive behaviours and hyperlocomotion with amygdalar NA levels and between non social behaviours and amygdalar GABA amount in BTBR and BL6 mice. **A** Correlation between poking holes (nr) and amygdalar NA (fmol/mg of tissue). Pearson correlation, *r* = −0.8857; **P = 0.0015; **B** Correlation between distance travelled (mt) and amygdalar NA (fmol/mg of tissue). Pearson correlation, *r* = −0.8608; **P = 0.0029; **C** Correlation between non social behaviours frequency (nr) and amygdalar GABA (uM/mg of tissue). Pearson correlation, *r* = −0.7203; *P = 0.0188.

In order to investigate the presence of correlations between ASD behaviours and ex vivo parameters in amygdala, we performed Pearson correlation in mice from both groups. We did not find any significant correlation (data not shown), except for the following reported results; we found a significant negative correlation between the number of poking holes in the Hole Board task and the levels of amygdalar NA (Fig. 5A Pearson correlation, *r* = −0.8857; *P* < 0.01), together with a significant negative correlation between the distance travelled in the Open Field test and the levels of amygdalar NA (Fig. 5B, Pearson correlation, *r* = −0.8608; *P* < 0.01). Moreover, frequency of non-social behaviours in the Social Interaction test and GABA levels in AMY were also negatively correlated (Fig. 5E, Pearson correlation, *r* = −0.7203; *P* < 0.05).

## Discussion

In the present study, the BTBR mice model reported ASD-like behavioural features, accompanied by several neurochemical alterations, in particular concerning the amygdalar area.

In this regard, we characterized the BTBR strain from a neurochemical point of view, by analysing dopaminergic, noradrenergic and cholinergic, together with excitatory-inhibitory neurotransmissions and NGF and BDNF expression levels, in four different brain areas that are involved in ASD and that are strictly interplayed among each other. As regards PFC, our results showed a decrease in NA, ACh and GABA levels, while the HIPP was characterized by enhanced DA metabolism and reduced NGF, BDNF and GABA content. Concerning HYP, no differences were retrieved among the different neurotransmitters analysed. Interestingly, we found significant alterations in the AMY, in which there was a decrease in NA, DA, ACh, GABA and NGF levels, together with an increase in DA metabolites and glutamate content, thus denoting massive dysfunctions in this brain region. According to our results, it has been reported that a significant activation of the DA turnover in AMY exacerbated the ASD-like behaviours in BTBR mice [66] and that a reduction in the DA release in AMY was associated with an enhancement in grooming episodes [67]. Moreover, it has been reported that impairment during AMY development is a crucial component of the neurophenotype of ASD, occurring certain time before behavioural diagnosis of ASD can reliably be made [68]. In addition, the increased AMY growth in ASD children has been associated with an enhancement of social dysfunctions [68]. Furthermore, it has been demonstrated that lesions in the AMY altered fear response and reduced anxiety [69]. However, it has to be taken into account that the basolateral nucleus of the AMY sends projections to the PFC and HIPP [70] and that these three areas are in continuous crosstalk. Indeed, several studies reported a dopaminergic modulation in widespread forebrain areas of ASD patients [67, 71–76]. In regards to ACh content, we found a decrease in PFC and AMY according to neurochemical alterations in the cholinergic pathway observed in a *postmortem* study involving ASD patients [77]. Moreover, it has been reported that ACh increase could lead to an improvement of cognitive deficits in ASD and other neuropsychiatric disorders [78]. Interestingly, reduced ACh in the basal forebrain has been linked to decreased social interactions and social memory dysfunctions in mice [51]. Furthermore, we also found a decrease in cortical and amygdalar NA levels in BTBR mice. In this regard, it has been reported that stereotyped behaviours, considered as derivatives of poor adaptive behaviours, are principally mediated by the noradrenergic system and that the administration of an alpha-2-receptor agonist might be helpful to improve ASD behavioural dysfunctions [79]. In line with our results, another model of ASD, the Engrailed-2 knock-out (EN2KO) mice, also reported NA neurotransmission alterations in the ventral hindbrain [80]. In addition, it has been shown that the administration of atomoxetine, a NA reuptake inhibitor, could be effective in treating hyperactivity in children with ASD [81]. In addition, we found a significant negative correlation between the number of poking holes (in the Hole Board test) with the amount of NA in AMY. Moreover, the levels of NA in AMY were also negatively correlated with the distance travelled, thus denoting a significant inverse correlation between repetitive behaviours and hyperactivity with NA levels. Accordingly, an important role of NA in attention-deficit/hyperactivity disorders has been reported, therefore different treatments for hyperactivity aim to increase NA brain levels [82]. On the other hand, the neurobiology of stereotyped behaviours is still controversial [83]. However, it has been demonstrated that mice lacking NA expressed excessive grooming behaviour, a well-known repetitive behaviour [84], and that NA upregulation reduced both hyperactivity and stereotypic behaviours [44], hence suggesting that NA alterations might be crucially involved in the development of such behavioural features and that the targeted modulation of noradrenergic neurotransmission could result in improving stereotyped repertoire and hyperactivity.

Furthermore, different studies reported an interplay between NA neurotransmission and NGF expression [85–87]. In particular, it has been shown that, in cellular models, monoamine oxidase inhibitors increased NGF expression [87] and that NA can exert neuroprotective properties by inducing NGF expression [85]. In this regard, our results showed that BTBR animals had decreased NGF and BDNF expression levels in HIPP, a brain region in which such neurotrophins are mainly present [88]. Accordingly, hippocampal BDNF and NGF deficiency has been associated with ASD development [89, 90], however results are contrasting, since BDNF brain levels fluctuations have been reported in response to several known, such as brain areas and age, and unknown factors [91]. In addition, NGF levels were reduced also in AMY, a brain region in which NGF signalling has been linked to stress, reward and neuroprotection [92]. Moreover, NGF expression might be modulated also by the GABAergic system. Indeed, it has been demonstrated that NGF production is predominantly localized in GABAergic inhibitory neurons [93]. In our results, we also found impairments of excitatory-inhibitory neurotransmissions. In particular, we showed an increase in glutamate and a reduction in GABA levels in the AMY, while in PFC and HIPP we only reported a decrease in GABA content. Thus, BTBR animals demonstrated a perturbation of the excitatory-inhibitory balance, by showing disruptions in the PFC and HIPP and disequilibrium in favour of glutamate in AMY. These data corroborate with our previous studies in which BTBR housed in a semi-natural environment showed a decrease in GABA levels in AMY [94]. Accordingly, great interest is moving toward GABA involvement in sociability pathways. Indeed, it has been shown that a reduction in GABA functions in the basolateral AMY triggered social interaction dysfunctions [95]. Regarding glutamate, already in 2008 Fatemi [96] proposed the hyperglutamatergic theory of autism. Furthermore, the reduction in GABA levels in AMY has been associated with decreased sociability, especially considering that AMY is a crucial component of the social brain [95]. Concerning behavioural alterations, our results confirmed stereotyped repertoire, social interaction deficits and novelty-induced hyperlocomotion of BTBR mice [97–100], but also showed an increase of the time spent performing non-social exploration, together with the frequency of such behaviour. In this regard, the exploration of three objects non socially-related might resemble the absence of interests in people and in developing relationships, an important behavioural feature of ASD patients [101]. Moreover, we found a significant negative correlation between GABA amount in AMY and frequency of non-social behaviours, which could mimic the indifference and the lack of interest for the surrounding social stimuli often described in ASD patients [102]. Lastly, we also showed that BTBR animals display lower anxiety levels, which could also be interpreted as enhanced hyperactivity and impulsiveness, important traits typical of ASD patients [103].

In conclusion, our neurochemical characterization of the BTBR strain suggested that such idiopathic animal model might be useful to unravel neurobiological and neurochemical correlates of ASD behavioural dysfunctions, highlighting the important role of neurotransmitter alterations in a specific brain region, such as AMY, and strengthening that, beyond the well-known face validity, BTBR mice also exhibit a high-grade of construct validity.

### Supplementary information

Supplementary Figures

## Data Availability

The data that support the findings of this study are available from the corresponding author upon reasonable request.

## References

[1] Carbone E, Manduca A, Cacchione C, Vicari S, Trezza V (2021). Healing autism spectrum disorder with cannabinoids: a neuroinflammatory story. Neurosci Biobehav Rev.

[2] Guo YP, Commons KG (2017). Serotonin neuron abnormalities in the BTBR mouse model of autism. Autism Res.

[3] Murray MJ (2010). Attention-deficit/Hyperactivity Disorder in the context of Autism spectrum disorders. Curr Psychiatry Rep.

[4] Hodges H, Fealko C, Soares N (2020). Autism spectrum disorder: definition, epidemiology, causes, and clinical evaluation. Transl Pediatr.

[5] Zeidan J, Fombonne E, Scorah J, Ibrahim A, Durkin MS, Saxena S (2022). Global prevalence of autism: A systematic review update. Autism Res.

[6] Scattoni ML, Fatta LM, Micai M, Sali ME, Bellomo M, Salvitti T (2023). Autism spectrum disorder prevalence in Italy: a nationwide study promoted by the Ministry of Health. Child Adolesc Psychiatry Ment Health.

[7] Sacco R, Camilleri, N, Eberhardt, J, Umla-Runge, K, & Newbury-Birch, D. The Prevalence of Autism Spectrum Disorder in Europe. Autism Spectrum Disorders - Recent Advances and New Perspectives. IntechOpen*.* 2023. 10.5772/intechopen.108123.

[8] Samsam M, Ahangari R, Naser SA (2014). Pathophysiology of autism spectrum disorders: revisiting gastrointestinal involvement and immune imbalance. World J Gastroenterol.

[9] Napolitano A, Schiavi S, La Rosa P, Rossi-Espagnet MC, Petrillo S, Bottino F (2022). Sex differences in autism spectrum disorder: diagnostic, neurobiological, and behavioral features. Front Psychiatry.

[10] Grosso G, Galvano F, Marventano S, Malaguarnera M, Bucolo C, Drago F (2014). Omega-3 fatty acids and depression: scientific evidence and biological mechanisms. Oxid Med Cell Longev.

[11] Grosso G, Pajak A, Marventano S, Castellano S, Galvano F, Bucolo C (2014). Role of omega-3 fatty acids in the treatment of depressive disorders: a comprehensive meta-analysis of randomized clinical trials. PLoS One.

[12] Blanchard DC, Defensor EB, Meyza KZ, Pobbe RL, Pearson BL, Bolivar VJ (2012). BTBR T+tf/J mice: autism-relevant behaviors and reduced fractone-associated heparan sulfate. Neurosci Biobehav Rev.

[13] Wohr M, Roullet FI, Crawley JN (2011). Reduced scent marking and ultrasonic vocalizations in the BTBR T+tf/J mouse model of autism. Genes Brain Behav.

[14] Scattoni ML, Martire A, Cartocci G, Ferrante A, Ricceri L (2013). Reduced social interaction, behavioural flexibility and BDNF signalling in the BTBR T+ tf/J strain, a mouse model of autism. Behav Brain Res.

[15] Chao OY, Yunger R, Yang YM (2018). Behavioral assessments of BTBR T+Itpr3tf/J mice by tests of object attention and elevated open platform: Implications for an animal model of psychiatric comorbidity in autism. Behav Brain Res.

[16] Chao OY, Marron Fernandez de Velasco E, Pathak SS, Maitra S, Zhang H, Duvick L (2020). Targeting inhibitory cerebellar circuitry to alleviate behavioral deficits in a mouse model for studying idiopathic autism. Neuropsychopharmacology.

[17] Kas MJ, Penninx B, Sommer B, Serretti A, Arango C, Marston H (2019). A quantitative approach to neuropsychiatry: The why and the how. Neurosci Biobehav Rev.

[18] Pearson BL, Pobbe RL, Defensor EB, Oasay L, Bolivar VJ, Blanchard DC (2011). Motor and cognitive stereotypies in the BTBR T+tf/J mouse model of autism. Genes Brain Behav.

[19] McPheeters ML, Warren Z, Sathe N, Bruzek JL, Krishnaswami S, Jerome RN (2011). A systematic review of medical treatments for children with autism spectrum disorders. Pediatrics.

[20] Sharma A, Shaw SR (2012). Efficacy of risperidone in managing maladaptive behaviors for children with autistic spectrum disorder: a meta-analysis. J Pediatr Health Care.

[21] Aishworiya R, Valica T, Hagerman R, Restrepo B (2022). An update on psychopharmacological treatment of autism spectrum disorder. Neurotherapeutics.

[22] Whitehouse CM, Lewis MH (2015). Repetitive behavior in neurodevelopmental disorders: clinical and translational findings. Behav Anal.

[23] Langen M, Kas MJ, Staal WG, van Engeland H, Durston S (2011). The neurobiology of repetitive behavior: of mice. Neurosci Biobehav Rev.

[24] Kemper TL, Bauman M (1998). Neuropathology of infantile autism. J Neuropathol Exp Neurol.

[25] Haznedar MM, Buchsbaum MS, Wei TC, Hof PR, Cartwright C, Bienstock CA (2000). Limbic circuitry in patients with autism spectrum disorders studied with positron emission tomography and magnetic resonance imaging. Am J Psychiatry.

[26] Hollander E, Anagnostou E, Chaplin W, Esposito K, Haznedar MM, Licalzi E (2005). Striatal volume on magnetic resonance imaging and repetitive behaviors in autism. Biol Psychiatry.

[27] Zilbovicius M, Boddaert N, Belin P, Poline JB, Remy P, Mangin JF (2000). Temporal lobe dysfunction in childhood autism: a PET study. Positron emission tomography. Am J Psychiatry.

[28] Gandhi T, Lee CC (2020). Neural mechanisms underlying repetitive behaviors in rodent models of autism spectrum disorders. Front Cell Neurosci.

[29] Rubenstein JL (2010). Three hypotheses for developmental defects that may underlie some forms of autism spectrum disorder. Curr Opin Neurol.

[30] Theoharides TC, Athanassiou M, Panagiotidou S, Doyle R (2015). Dysregulated brain immunity and neurotrophin signaling in Rett syndrome and autism spectrum disorders. J Neuroimmunol.

[31] Barbosa AG, Pratesi R, Paz GSC, Dos Santos M, Uenishi RH, Nakano EY (2020). Assessment of BDNF serum levels as a diagnostic marker in children with autism spectrum disorder. Sci Rep.

[32] Crespi BJ (2019). Comparative psychopharmacology of autism and psychotic-affective disorders suggests new targets for treatment. Evol Med Public Health.

[33] Hellings JA, Arnold LE, Han JC (2017). Dopamine antagonists for treatment resistance in autism spectrum disorders: review and focus on BDNF stimulators loxapine and amitriptyline. Expert Opin Pharmacother.

[34] von Richthofen S, Lang UE, Hellweg R (2003). Effects of different kinds of acute stress on nerve growth factor content in rat brain. Brain Res.

[35] Lu AT, Yoon J, Geschwind DH, Cantor RM (2013). QTL replication and targeted association highlight the nerve growth factor gene for nonverbal communication deficits in autism spectrum disorders. Mol Psychiatry.

[36] Minnone G, De Benedetti F, Bracci-Laudiero L (2017). NGF and its receptors in the regulation of inflammatory response. Int J Mol Sci.

[37] Paredes D, Granholm AC, Bickford PC (2007). Effects of NGF and BDNF on baseline glutamate and dopamine release in the hippocampal formation of the adult rat. Brain Res.

[38] Furukawa Y, Furukawa S, Satoyoshi E, Hayashi K (1986). Catecholamines induce an increase in nerve growth factor content in the medium of mouse L-M cells. J Biol Chem.

[39] Sara SJ (2009). The locus coeruleus and noradrenergic modulation of cognition. Nat Rev Neurosci.

[40] Aston-Jones G (2005). Brain structures and receptors involved in alertness. Sleep Med.

[41] DiCarlo GE, Aguilar JI, Matthies HJ, Harrison FE, Bundschuh KE, West A (2019). Autism-linked dopamine transporter mutation alters striatal dopamine neurotransmission and dopamine-dependent behaviors. J Clin Invest.

[42] Mandic-Maravic V, Grujicic R, Milutinovic L, Munjiza-Jovanovic A, Pejovic-Milovancevic M (2021). Dopamine in Autism Spectrum Disorders-Focus on D2/D3 partial agonists and their possible use in treatment. Front Psychiatry.

[43] Paval D, Miclutia IV (2021). The Dopamine hypothesis of autism spectrum disorder revisited: current status and future prospects. Dev Neurosci.

[44] Beversdorf DQ (2020). The role of the noradrenergic system in autism spectrum disorders, implications for treatment. Semin Pediatr Neurol.

[45] Leshem R, Bar-Oz B, Diav-Citrin O, Gbaly S, Soliman J, Renoux C (2021). Selective Serotonin Reuptake Inhibitors (SSRIs) and Serotonin Norepinephrine Reuptake Inhibitors (SNRIs) During Pregnancy and the Risk for Autism spectrum disorder (ASD) and Attention deficit hyperactivity disorder (ADHD) in the Offspring: A True Effect or a Bias? A Systematic Review & Meta-Analysis. Curr Neuropharmacol.

[46] Kubota M, Fujino J, Tei S, Takahata K, Matsuoka K, Tagai K (2020). Binding of Dopamine D1 receptor and Noradrenaline transporter in individuals with autism spectrum disorder: A PET study. Cereb Cortex.

[47] Yizhar O, Fenno LE, Prigge M, Schneider F, Davidson TJ, O’Shea DJ (2011). Neocortical excitation/inhibition balance in information processing and social dysfunction. Nature.

[48] Blatt GJ, Fatemi SH (2011). Alterations in GABAergic biomarkers in the autism brain: research findings and clinical implications. Anat Rec.

[49] Horder J, Petrinovic MM, Mendez MA, Bruns A, Takumi T, Spooren W (2018). Glutamate and GABA in autism spectrum disorder-a translational magnetic resonance spectroscopy study in man and rodent models. Transl Psychiatry.

[50] Tanimizu T, Kenney JW, Okano E, Kadoma K, Frankland PW, Kida S (2017). Functional connectivity of multiple brain regions required for the consolidation of social recognition memory. J Neurosci.

[51] Kljakic O, Al-Onaizi M, Janickova H, Chen KS, Guzman MS, Prado MAM (2021). Cholinergic transmission from the basal forebrain modulates social memory in male mice. Eur J Neurosci.

[52] Sato W, Uono S (2019). The atypical social brain network in autism: advances in structural and functional MRI studies. Curr Opin Neurol.

[53] Odriozola P, Dajani DR, Burrows CA, Gabard-Durnam LJ, Goodman E, Baez AC (2019). Atypical frontoamygdala functional connectivity in youth with autism. Dev Cogn Neurosci.

[54] Fishman I, Linke AC, Hau J, Carper RA, Muller RA (2018). Atypical functional connectivity of amygdala related to reduced symptom severity in children with autism. J Am Acad Child Adolesc Psychiatry.

[55] Bellani M, Calderoni S, Muratori F, Brambilla P (2013). Brain anatomy of autism spectrum disorders II. Focus on amygdala. Epidemiol Psychiatr Sci.

[56] Bellani M, Calderoni S, Muratori F, Brambilla P (2013). Brain anatomy of autism spectrum disorders I. Focus on corpus callosum. Epidemiol Psychiatr Sci.

[57] Pardo CA, Eberhart CG (2007). The neurobiology of autism. Brain Pathol.

[58] Lee JK, Andrews DS, Ozturk A, Solomon M, Rogers S, Amaral DG (2022). Altered development of Amygdala-connected brain regions in males and females with autism. J Neurosci.

[59] Loomes R, Hull L, Mandy WPL (2017). What is the male-to-female ratio in autism spectrum disorder? A systematic review and meta-analysis. J Am Acad Child Adolesc Psychiatry.

[60] Souza LS, Silva EF, Santos WB, Asth L, Lobao-Soares B, Soares-Rachetti VP (2016). Lithium and valproate prevent methylphenidate-induced mania-like behaviors in the hole board test. Neurosci Lett.

[61] Carola V, D’Olimpio F, Brunamonti E, Mangia F, Renzi P (2002). Evaluation of the elevated plus-maze and open-field tests for the assessment of anxiety-related behaviour in inbred mice. Behav Brain Res.

[62] Bove M, Schiavone S, Tucci P, Sikora V, Dimonte S, Colia AL (2022). Ketamine administration in early postnatal life as a tool for mimicking Autism Spectrum Disorders core symptoms. Prog Neuropsychopharmacol Biol Psychiatry.

[63] Kraeuter AK, Guest PC, Sarnyai Z (2019). Free dyadic social interaction test in mice. Methods Mol Biol.

[64] Laviola G, Adriani W, Rea M, Aloe L, Alleva E (2004). Social withdrawal, neophobia, and stereotyped behavior in developing rats exposed to neonatal asphyxia. Psychopharmacol (Berl).

[65] Tucker LB, McCabe JT (2017). Behavior of male and female C57BL/6J mice is more consistent with repeated trials in the elevated zero maze than in the elevated plus maze. Front Behav Neurosci.

[66] Pascucci T, Colamartino M, Fiori E, Sacco R, Coviello A, Ventura R (2020). P-cresol alters brain dopamine metabolism and exacerbates autism-like behaviors in the BTBR mouse. Brain Sci.

[67] Squillace M, Dodero L, Federici M, Migliarini S, Errico F, Napolitano F (2014). Dysfunctional dopaminergic neurotransmission in asocial BTBR mice. Transl Psychiatry.

[68] Shen MD, Swanson MR, Wolff JJ, Elison JT, Girault JB, Kim SH (2022). Subcortical brain development in autism and fragile X Syndrome: Evidence for dynamic, age- and disorder-specific trajectories in infancy. Am J Psychiatry.

[69] Korn CW, Vunder J, Miro J, Fuentemilla L, Hurlemann R, Bach DR (2017). Amygdala lesions reduce anxiety-like behavior in a human Benzodiazepine-sensitive approach-avoidance conflict test. Biol Psychiatry.

[70] Obeso JA, Lanciego JL (2011). Past, present, and future of the pathophysiological model of the Basal Ganglia. Front Neuroanat.

[71] Nguyen M, Roth A, Kyzar EJ, Poudel MK, Wong K, Stewart AM (2014). Decoding the contribution of dopaminergic genes and pathways to autism spectrum disorder (ASD). Neurochem Int.

[72] Ghanizadeh A, Moghimi-Sarani E (2013). A randomized double blind placebo controlled clinical trial of N-Acetylcysteine added to risperidone for treating autistic disorders. BMC Psychiatry.

[73] Chadman KK, Guariglia SR, Yoo JH (2012). New directions in the treatment of autism spectrum disorders from animal model research. Expert Opin Drug Discov.

[74] Martineau J, Roux S, Adrien JL, Garreau B, Barthelemy C, Lelord G (1992). Electrophysiological evidence of different abilities to form cross-modal associations in children with autistic behavior. Electroencephalogr Clin Neurophysiol.

[75] Launay JM, Bursztejn C, Ferrari P, Dreux C, Braconnier A, Zarifian E (1987). Catecholamines metabolism in infantile autism: a controlled study of 22 autistic children. J Autism Dev Disord.

[76] Garnier C, Comoy E, Barthelemy C, Leddet I, Garreau B, Muh JP (1986). Dopamine-beta-hydroxylase (DBH) and homovanillic acid (HVA) in autistic children. J Autism Dev Disord.

[77] Marotta R, Risoleo MC, Messina G, Parisi L, Carotenuto M, Vetri L (2020). The neurochemistry of autism. Brain Sci.

[78] Wang L, Almeida LE, Spornick NA, Kenyon N, Kamimura S, Khaibullina A (2015). Modulation of social deficits and repetitive behaviors in a mouse model of autism: the role of the nicotinic cholinergic system. Psychopharmacology.

[79] Nanjappa MS, Voyiaziakis E, Pradhan B, Mannekote Thippaiah S (2022). Use of selective serotonin and norepinephrine reuptake inhibitors (SNRIs) in the treatment of autism spectrum disorder (ASD), comorbid psychiatric disorders and ASD-associated symptoms: a clinical review. CNS Spectr.

[80] Brielmaier J, Senerth JM, Silverman JL, Matteson PG, Millonig JH, DiCicco-Bloom E (2014). Chronic desipramine treatment rescues depression-related, social and cognitive deficits in Engrailed-2 knockout mice. Genes Brain Behav.

[81] Arnold LE, Aman MG, Cook AM, Witwer AN, Hall KL, Thompson S (2006). Atomoxetine for hyperactivity in autism spectrum disorders: placebo-controlled crossover pilot trial. J Am Acad Child Adolesc Psychiatry.

[82] Del Campo N, Chamberlain SR, Sahakian BJ, Robbins TW (2011). The roles of dopamine and noradrenaline in the pathophysiology and treatment of attention-deficit/hyperactivity disorder. Biol Psychiatry.

[83] Langen M, Durston S, Kas MJ, van Engeland H, Staal WG (2011). The neurobiology of repetitive behavior: …and men. Neurosci Biobehav Rev.

[84] Lustberg DJ, Liu JQ, Iannitelli AF, Vanderhoof SO, Liles LC, McCann KE (2022). Norepinephrine and dopamine contribute to distinct repetitive behaviors induced by novel odorant stress in male and female mice. Horm Behav.

[85] Counts SE, Mufson EJ (2010). Noradrenaline activation of neurotrophic pathways protects against neuronal amyloid toxicity. J Neurochem.

[86] Benitez A, Riquelme R, Del Campo M, Araya C, Lara HE (2021). Nerve growth factor: a dual activator of noradrenergic and cholinergic systems of the rat ovary. Front Endocrinol (Lausanne).

[87] Naoi M, Maruyama W (2010). Monoamine oxidase inhibitors as neuroprotective agents in age-dependent neurodegenerative disorders. Curr Pharm Des.

[88] Dincel N, Unalp A, Kutlu A, Ozturk A, Uran N, Ulusoy S (2013). Serum nerve growth factor levels in autistic children in Turkish population: a preliminary study. Indian J Med Res.

[89] Dingsdale H, Garay SM, Tyson HR, Savory KA, Sumption LA, Kelleher JS (2022). Cord serum brain-derived neurotrophic factor levels at birth associate with temperament outcomes at one year. J Psychiatr Res.

[90] Liu C, Liu J, Gong H, Liu T, Li X, Fan X (2023). Implication of Hippocampal Neurogenesis in autism spectrum disorder: pathogenesis and therapeutic implications. Curr Neuropharmacol.

[91] Kasarpalkar NJ, Kothari ST, Dave UP (2014). Brain-derived neurotrophic factor in children with autism spectrum disorder. Ann Neurosci.

[92] Meis S, Endres T, Lessmann V (2020). Neurotrophin signalling in amygdala-dependent cued fear learning. Cell Tissue Res.

[93] Biane J, Conner JM, Tuszynski MH (2014). Nerve growth factor is primarily produced by GABAergic neurons of the adult rat cortex. Front Cell Neurosci.

[94] Bove M, Ike K, Eldering A, Buwalda B, de Boer SF, Morgese MG (2018). The Visible Burrow System: A behavioral paradigm to assess sociability and social withdrawal in BTBR and C57BL/6J mice strains. Behav Brain Res.

[95] Paine TA, Swedlow N, Swetschinski L (2017). Decreasing GABA function within the medial prefrontal cortex or basolateral amygdala decreases sociability. Behav Brain Res.

[96] Fatemi SH (2008). The hyperglutamatergic hypothesis of autism. Prog Neuropsychopharmacol Biol Psychiatry.

[97] Brierley NJ, McDonnell CG, Parks KMA, Schulz SE, Dalal TC, Kelley E (2021). Factor structure of repetitive behaviors across autism spectrum disorder and attention-deficit/hyperactivity disorder. J Autism Dev Disord.

[98] McFarlane HG, Kusek GK, Yang M, Phoenix JL, Bolivar VJ, Crawley JN (2008). Autism-like behavioral phenotypes in BTBR T+tf/J mice. Genes Brain Behav.

[99] Moy SS, Nadler JJ, Young NB, Perez A, Holloway LP, Barbaro RP (2007). Mouse behavioral tasks relevant to autism: phenotypes of 10 inbred strains. Behav Brain Res.

[100] Silverman JL, Tolu SS, Barkan CL, Crawley JN (2010). Repetitive self-grooming behavior in the BTBR mouse model of autism is blocked by the mGluR5 antagonist MPEP. Neuropsychopharmacology.

[101] Davidson C, O’Hare A, Mactaggart F, Green J, Young D, Gillberg C (2015). Social relationship difficulties in autism and reactive attachment disorder: Improving diagnostic validity through structured assessment. Res Dev Disabil.

[102] Haworth J, Kyvelidou A, Fisher W, Stergiou N (2016). Indifference to chaotic motion may be related to social disinterest in children with autism. J Mot Learn Dev.

[103] Rodrigues R, Lai MC, Beswick A, Gorman DA, Anagnostou E, Szatmari P (2021). Practitioner Review: Pharmacological treatment of attention-deficit/hyperactivity disorder symptoms in children and youth with autism spectrum disorder: a systematic review and meta-analysis. J Child Psychol Psychiatry.

