# Supplementary material for: Amygdalar neurotransmission alterations in the BTBR mice model of idiopathic autism
Source: Transl Psychiatry. 2024 Apr 17;14:193. doi: 10.1038/s41398-024-02905-z (PMC11024334; doi:10.1038/s41398-024-02905-z)
Supplement: Supplementary file 1 — Supplementary Figures [file 41398_2024_2905_MOESM1_ESM.pdf]

Supplementary figure 1

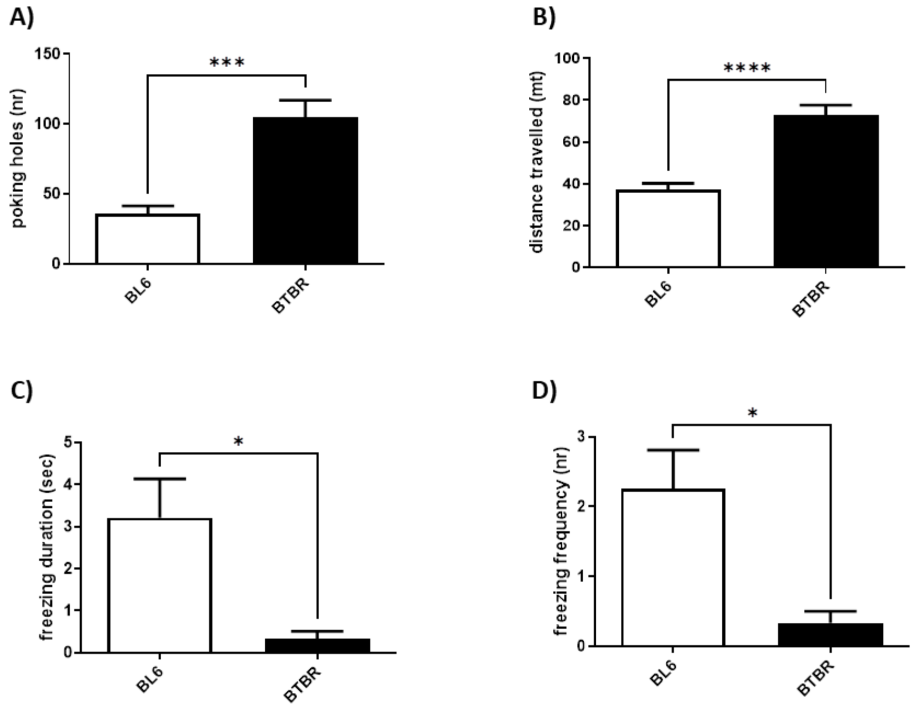

Supplementary figure 1. *Number of Poking Holes in the Hole Board Task and distance travelled, freezing duration and freezing frequency in the Open Field test of BTBR (black bar) and BL6 (white bar) mice.*

*A) Number of poking holes (nr) in BL6 (n = 8) and BTBR (n = 7) mice. Unpaired Student's t-test, ***P < 0.001 BTBR vs. BL6; B) Distance travelled (mt) for BL6 (n = 8) and BTBR (n = 9) mice. Unpaired Student's t-test, ****P < 0.0001 BTBR vs. BL6; C) Duration of freezing (sec) for BL6 (n = 8) and BTBR (n = 9) mice. Unpaired Student's t-test, *P < 0.05 BTBR vs. BL6; D) Freezing frequency (n) in BL6 (n = 8) and BTBR (n = 9) mice. Unpaired Student's t-test, *P < 0.05 BTBR vs. BL6.*

Supplementary figure 2

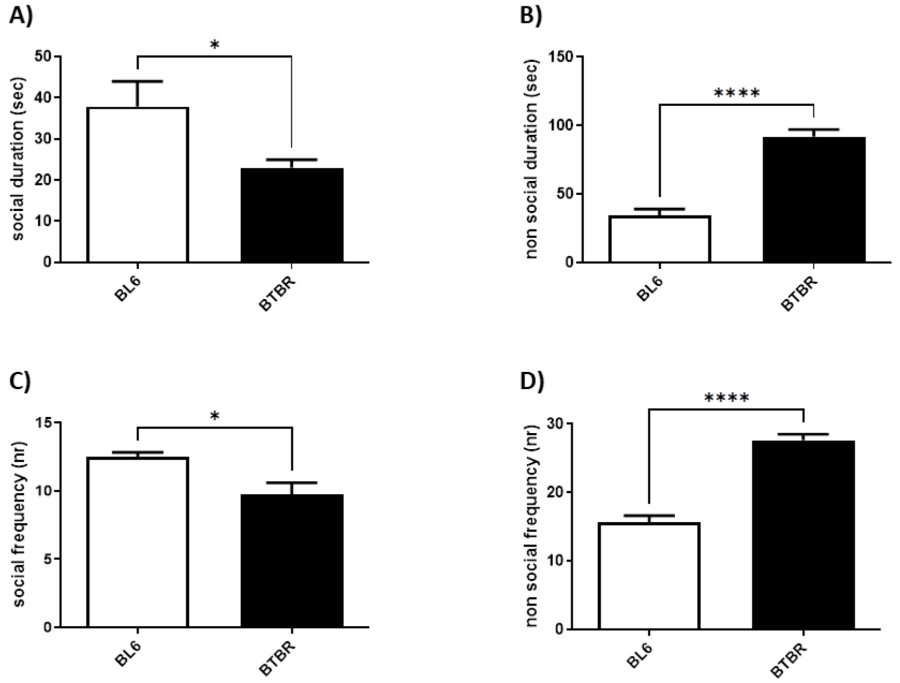

Supplementary figure 2. *Social and non-social duration and social and non-social frequency in the Social Interaction test of BTBR (black bar) and BL6 (white bar) mice.*

*A) Social duration (sec) in BL6 (n = 10) and BTBR (n = 8) mice. Unpaired Student's t-test, *P < 0.05 BTBR vs. BL6; B) Non-social duration (sec) in BL6 (n = 10) and BTBR (n = 8) mice. Unpaired Student's t-test, ****P < 0.0001 BTBR vs. BL6; C) Social frequency (n) in BL6 (n = 10) and BTBR (n = 8) mice. Unpaired Student's t-test, *P < 0.05 BL6 vs. BTBR; D) Non-social frequency (n) in BL6 (n = 10) and BTBR (n = 8) mice. Unpaired Student's t-test, ****P < 0.0001 BTBR vs. BL6.*

Supplementary figure 3

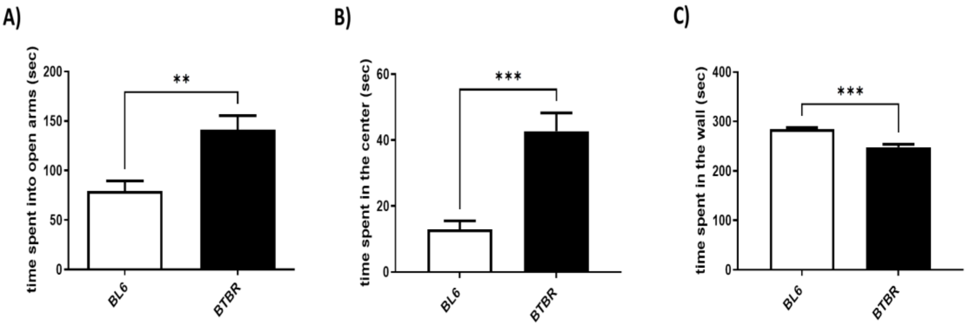

Supplementary figure 3. *Time spent into open and closed arms in the Elevated Zero Maze test and in the center and the wall in Open Field test of BTBR (black bar) and BL6 (white bar) mice.*

*A) Time (sec) spent into open arms in BL6 (n = 7) and BTBR (n = 7) mice. Unpaired Student's t-test, **P < 0.01 BTBR vs. BL6; B) Time (sec) spent in the center in BL6 (n = 8) and BTBR (n = 9) mice. Unpaired Student's t-test, ***P < 0.001 BTBR vs. BL6; C) Time (sec) spent in the wall in BL6 (n = 8) and BTBR (n = 9) mice. Unpaired Student's t-test, ***P < 0.001 BTBR vs. BL6.*
